# Atypical Prolonged Viral Shedding With Intra-Host SARS-CoV-2 Evolution in a Mildly Affected Symptomatic Patient

**DOI:** 10.3389/fmed.2021.760170

**Published:** 2021-11-26

**Authors:** Marielton dos Passos Cunha, Ana Paula Pessoa Vilela, Camila Vieira Molina, Stephanie Maia Acuña, Sandra Marcia Muxel, Vinícius de Morais Barroso, Sabrina Baroni, Lilian Gomes de Oliveira, Yan de Souza Angelo, Jean Pierre Schatzmann Peron, Luiz Gustavo Bentim Góes, Angélica Cristine de Almeida Campos, Paola Minóprio

**Affiliations:** ^1^Scientific Platform Pasteur—USP, São Paulo, Brazil; ^2^Institute of Biomedical Sciences, University of São Paulo, São Paulo, Brazil; ^3^Institut Pasteur, Paris, France

**Keywords:** SARS-CoV-2, prolonged RNA viral shedding, immune response, coronavirus, intra-host evolution

## Abstract

The severe acute respiratory syndrome coronavirus 2 (SARS-CoV-2) infection is caused by a respiratory virus with a wide range of manifestations, varying from asymptomatic to fatal cases, with a generally short outcome. However, some individuals present long-term viral shedding. We monitored 38 individuals who were mildly affected by the SARS-CoV-2 infection. Out of the total studied population, three (7.9%) showed atypical events regarding the duration of positivity for viral RNA detection. In one of these atypical cases, a previously HIV-positive male patient presented a SARS-CoV-2 RNA shedding and subgenomic RNA (sgRNA) detected from the upper respiratory tract, respectively, for 232 and 224 days after the onset of the symptoms. The SARS-CoV-2 B.1.1.28 lineage, one of the most prevalent in Brazil in 2020, was identified in this patient in three serial samples. Interestingly, the genomic analyses performed throughout the infectious process showed an increase in the genetic diversity of the B.1.1.28 lineage within the host itself, with viral clearance occurring naturally, without any intervention measures to control the infection. Contrasting widely spread current knowledge, our results indicate that potentially infectious SARS-CoV-2 virus might be shed by much longer periods by some infected patients. This data call attention to better adapted non-pharmacological measures and clinical discharge of patients aiming at preventing the spread of SARS-CoV-2 to the population.

## Introduction

The severe acute respiratory syndrome coronavirus 2 (SARS-CoV-2) emerged in Wuhan, Hubei province, China, in late December 2019 (1–3) causing highly transmissible respiratory infection and acute disease in humans. The SARS-CoV-2 (family *Coronaviridae*, genus *Betacoronavirus*) quickly spread over a large geographical area (4), impacting a global scale in terms of morbidity, mortality, and economic impact. The first diagnosed SARS-CoV-2 patient in Brazil occurred on February 26, 2020 (5). The virus rapidly started its community circulation process caused by multiple lineages in a short period in all Brazilian states (6), and distinct viral lineages were identified (6–8).

Since the SARS-CoV-2 emergence, hundreds of thousands of viral consensus genomes have been sequenced and quickly made available around the world (9). The rapid spread of SARS-CoV-2, initially with an apparent low diversity, suggested that the ancestor of the virus accumulated a genetic diversity allowing for the emergence of several phylogenetic lineages while spreading geographically across the world (9). Recently, genetically diverse viral populations that co-circulate within a single host began to be explored in some population groups infected with SARS-CoV-2 (10–13).

Generally, SARS-CoV-2 causes acute respiratory syndrome with a rapid and broad clinical outcome. However, some studies had reported viral RNA (vRNA) and infectious particles for an atypical considered period (14–16). Other studies that characterized the intra-host viral diversity in patients with persistent infection showed a strong selection bias, with the introduction of neutralizing antibody cocktails or several interventions to treat secondary symptoms to the virus action (11, 12). Here, we explore the clinical and molecular aspects of the prolonged viral shedding process in a patient with evidence of viral replication for extended period. An intra-host genomic evolution process was evidenced by the detection of subgenomic RNA (sgRNA) and by the accumulated viral mutations over the period.

## Materials and Methods

### Ethical Statement

The samples analyzed in this study were collected after the consent of the patient following the protocol approved by the Human Research Ethics Committee of the Biomedical Sciences Institute—University of São Paulo (CEPSH-ICB-USP) (no. 4.036.252).

### Patients and Samples

This study is a molecular investigation of symptomatic, mildly affected patients, sampled between April 2020 and November 2020 in the city of São Paulo. In total, 721 patients with symptoms were screened for the detection of viral RNA of SARS-CoV-2. This first criterion of inclusion consisted in the presence of cold/flu symptoms, such as fever and/or respiratory symptoms (cough, breath shortness, and sore throat), following criteria recommended by the Brazilian Ministry of Health guidelines for Coronavirus Disease 2019 (COVID-19) diagnosis and treatment. Exclusion criteria included patients presenting symptoms of viral infections related to other agents than SARS-CoV-2, according to the same guidelines. *Thirty-eight* mild symptomatic positive to the SARS-CoV-2 detection out of 721 symptomatic patients were included in the present work. For inclusion in this group, the criteria were the acceptance to be followed weekly from the first episode of molecular positivity to the SARS-CoV-2 infection until they completed at least two or three consecutive episodes of negativity by reverse transcription-quantitative polymerase chain reaction (RT-qPCR) in nasopharyngeal-oropharyngeal (NP-OP) swab samples used for the screening and viral molecular diagnosis. Blood of all patients was collected individually after the first episode of molecular positivity for further serological analyses.

### Molecular Characterization

Nucleic acid extraction from all the NP-OP swabs was performed using the MagMAX™ Viral/Pathogen II (MVP II) Nucleic Acid Isolation Kit (Catalog number: A48383; Applied Biosystems, Waltham, MA, USA) and carried out according to the instructions of the manufacturer. Molecular detection of SARS-CoV-2 was performed using the AgPath-ID One-Step RT-PCR Reagents (Ambion, Austin, TX, USA). For the first screening, specific SARS-CoV-2 primers and probes were applied to the Envelope (E) gene, followed by the detection of the RNA-dependent RNA polymerase (RdRp) gene (17), as recommended by the WHO (18, 19). The screening study included some patients who were positive for the SARS-CoV-2 E gene but were considered as having an inconclusive diagnosis for COVID-19 due to negative results for the RdRp gene. They were followed up according to the same criteria used for confirmed cases (Supplementary Table 1). To detect sgRNA, we applied one of the Envelope primer and the probe in combination with other primer previously described (20). The RT-qPCR reactions consisted of a step of reverse transcription at 45°C for 10 min, enzyme activation at 95°C for 10 min, and 50 cycles at 95°C for 15 sec, and 60°C for 45 sec for hybridization and extension using QuantStudio™ 3 Real-Time PCR System (Thermo Fisher Scientific Inc., Waltham, MA, USA) to collect a fluorescence signal at the end of each cycle. We used the isolated virus SP02/BRA as the positive control, which was kindly provided by the Laboratory of Clinical Virology, Institute of Biomedical Sciences, University of São Paulo (21).

### Serological Characterization

We assessed patients anti-SARS-CoV-2 antibody production performing an enzyme-linked immunosorbent assay (ELISA), using sera of positive patients to analyze the presence of specific IgA and IgG against the viral nucleocapsid C-terminal portion, which was kindly provided by Prof. L. C. S. Ferreira (Institute of Biomedical Sciences, University of São Paulo, São Paulo, Brazil).

MaxiSorp plates (Nalge Nunc International, Rochester, NY, USA) were coated with antigen (375 ng in 50 μl of 1 × phosphate-buffered saline [PBS] per well) and incubated overnight at 4°C. We used 1 × PBS plus 2.5% of heath inactivated Fetal Bovine Serum as a blocking buffer (PBS-FBS −200 μl/well), incubating for 1 h at room temperature (RT). After, the diluted sera (1:100 in a PBS-FBS) was added (50 μl/well) and incubated at RT for 2 h. Subsequently, secondary peroxidase-conjugated anti-IgA (1:16,000 in PBS-FBS; Sigma Aldrich, St Luis, MO, USA) or anti-IgG (1:8,000 in PBS-FBS; Sigma Aldrich, St Luis, MO, USA) antibodies were added to each well (75 μl/well) and incubated at 37°C for 50 min. To reveal the reaction, 100 μl/well of the reagent 3,3′,5,5′-TetraMethylBenzidine (TMB) (Thermo Fisher Scientific Inc., Waltham, MA, USA) were added and incubated in the dark for 10 min at RT. Then, the enzymatic reaction was stopped using 100 μl/well of 0.2 N H_2_SO_4_. Between the blocking steps and the enzymatic reaction, four washing steps were performed using 1 × PBS with 0.05% Tween 20. We used a Multiskan™ FC Microplate Photometer (Thermo Fisher Scientific Inc., Waltham, MA, USA) to read the reaction, considering the difference between the optical density (OD) at 450 and 620 nm.

### Receptor-Binding Inhibition Assay (Surrogate Virus Neutralization Test Kit, SVNT)

The ability of the patient to neutralize SARS-CoV-2 was assessed through a sVNT (Catalog number: L00847-A; GenScript, Piscataway, NJ, USA), following manufacturer instructions (22). Their sera were incubated with a recombinant receptor-binding domain conjugated with horseradish peroxidase (RBD-HRP) at the final dilution of 1:20 for 30 min at 37°C. Afterward, the combination was added to a plate pre-coated with human ACE2 (hACE2) and incubated for 15 min at 37°C. Unbound RBD-HRP was removed by four washes, using the provided washing solution. The colorimetric signal was developed on the enzymatic reaction of HRP with 100 μl with TMB. Then, 50 μl of provided stop solution was added, and the OD at 450 nm using Multiskan™ FC Microplate Photometer (Thermo Fisher Scientific Inc., Waltham, MA, USA). Percentage of inhibition (%) = (1—Sample OD value/Negative Control OD value) × 100. The sVNT inhibition was settled as positive when the percentage value resulted in inhibition ≥ 20% (98.9% sensitivity and 100% specificity), as previously validated (22).

### Virus Isolation

Samples with positive RT-qPCR NP-OP were submitted to viral isolation. To do this, Vero CCL81 cells were seeded in 24-well plates and incubated overnight at 37°C, using a 5% CO_2_ incubator. The initial inoculum (passage 1) was prepared to dilute the clinical sample (1:5) in non-supplemented Dulbecco's Modified Eagle Medium (DMEM) low glucose media (LGC Biotecnologia, São Paulo, Brazil). The inoculum was then added to the monolayer cells, homogenized, and maintained for 1 h at 37°C. The inoculum was then removed, cells were washed two times with warm PBS and fresh DMEM low glucose media supplemented with FBS (2%) and penicillin/streptomycin (1%, 10,000 U/ml) was reloaded. The cell culture was observed for a cytopathic effect every day after the inoculation. Seventy-two-hour post-infection the cell culture supernatant was collected, centrifuged for the removal of debris (500 g, 10 min, 4°C) and stored at −80°C as the first viral passage. The same strategy was employed three times to obtain a third isolate passage. To confirm the isolation, we submitted the supernatants of each passage to RT-qPCR molecular assay to detect the Envelope gene, as mentioned above. Viral samples were considered isolated when the Ct value has dropped between passages one and three. We considered samples with Ct below 35 suitable for the next isolation passages. Samples in which the Ct value has not dropped were considered as non-isolated samples.

### Sequencing and Viral Genome Assembly

Based on the Ct values of the NP-OP samples, the total RNA was extracted using the MagMAX™ Viral/Pathogen II (MVP II) Nucleic Acid Isolation Kit (Catalog number: A48383) (Applied Biosystems) and carried out according to the instructions of the manufacturer. The target whole viral genome library preparation was constructed using the QIAseq^TM^ SARS-CoV-2 Primer Panel (Catalog number: 333896; Qiagen, Hilden, Germany). Sequencing was done at the Core Facility to Support Research—University of São Paulo (CEFAP-USP/GENIAL) using the Illumina MiSeq platform. Each sample was barcoded individually, which allowed the separation of reads for each one of them. Short unpaired reads and low-quality bases and reads were removed using Trimmomatic version 0.39 (LEADING:20 TRAILING:20 SLIDINGWINDOW:4:25 MINLEN:36) (23). Consensus genomes were assembled with paired end reads using Bowtie2 version 2.0.6 using default parameters (24). All the datasets analyzed during the current study are available from the corresponding author on reasonable request. The new sequence here characterized was deposited in GenBank under the accession number MW495017.

### SARS-CoV-2 Lineage Nomenclature and Phylogenetic Analysis

The viral genome sequences obtained from the NP-OP samples and assembled in this study were submitted to the SARS-CoV-2 lineage assignment using the Phylogenetic Assignment of Named Global Outbreak LINeages web application following a methodology previously described (9). Phylogenetic tree of SARS-CoV-2 based on full-length, curated sequences was estimated using the Maximum Likelihood (ML) method implemented in IQ-TREE 1.5.5 (25) with automatic model selection by ModelFinder and using the Bayesian Information Criterion (BIC) (26). To characterize the sequences of the atypical patient studied, we retrieved all complete sequences in GISAID (June 28, 2021), collected in Brazil for SARS-CoV-2 lineage B.1.1.28 with high coverage and with collection date completed. In total, 1,303 sequences were retrieved (Supplementary Figure 1). Sequences were initially resampled, excluding all identical sequences (https://biopython.org/wiki/Sequence_Cleaner), and after separation by Brazilian states, the outliers were resampled considering the average of the low sampled Brazilian states + 2 x SD (n = 53 sequences; https://web.expasy.org/decrease_redundancy/). In the end, we worked with a final dataset containing 56 sequences, 53 from São Paulo state and 2 sequences from the atypical patient, and we included the reference genome as an outgroup (NC_045512.2). The robustness of the groupings observed was assessed using 1,000 non-parametric bootstrap replicates. ML tree was visualized and plotted using FigTree v.1.4.3 (27). All taxon labels for sequences used in this work are presented in the format: hCoV-19/local of isolation/strain name/date of isolation.

### Statistical Analysis

The Shapiro–Wilk normality test was used in the present study. To compare the differences in the duration of positivity and symptoms according to each biological sex, the Mann–Whitney *U* test was applied, assuming a significance level of 0.05. All the statistical analysis and most of the graphs presented here were performed using R scripts and are available upon request.

## Results

**Figure 1:**
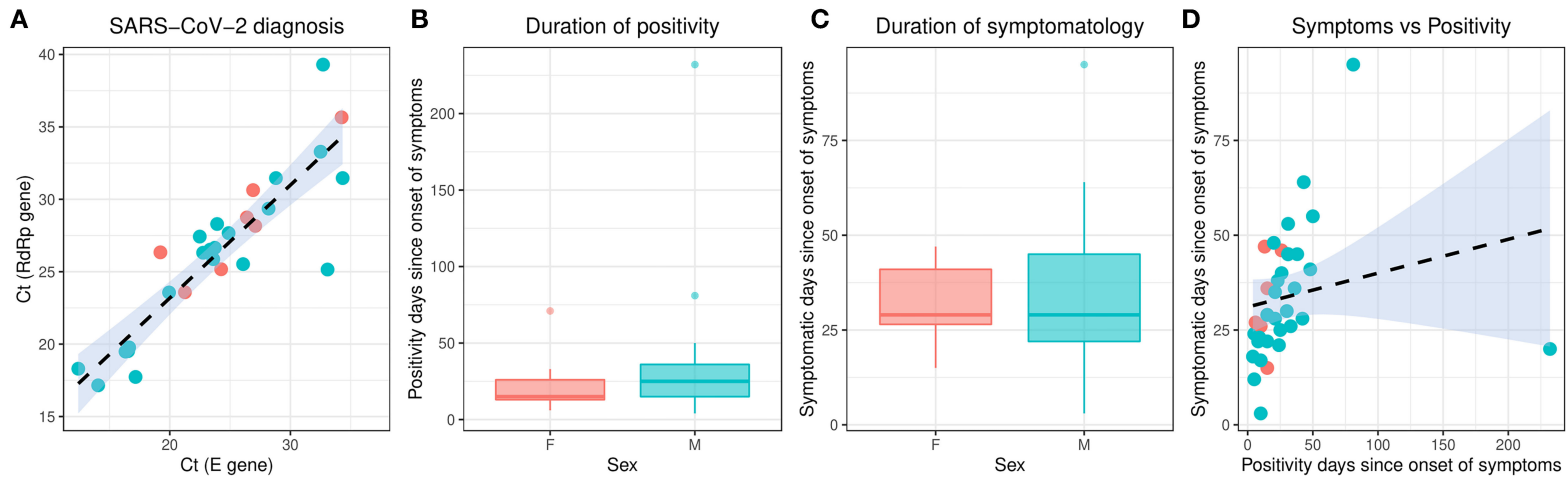
Molecular characterization of the studied individuals during the first SARS-CoV-2 pandemic phase in Brazil. The different colors represent the female and male individuals. **(A)** Correlation of the SARS-CoV-2 detection to Envelope and RdRp genes. **(B)** Duration of positivity on Envelope molecular assay after the symptom onset. Boxplots represent the 75th percentile, median, 25th percentile, and the whiskers extend to the highest and lowest value in the 1.5 × interquartile range. **(C)** Duration of symptomatology since the onset of symptoms. Boxplots represent the 75th percentile, median, 25th percentile, and the whiskers extend to the highest and lowest value in the 1.5 × interquartile range. **(D)** Correlation of the duration of symptoms to the duration of positivity after the onset of symptoms. SARS-CoV-2, severe acute respiratory syndrome coronavirus 2.

**Figure 2:**
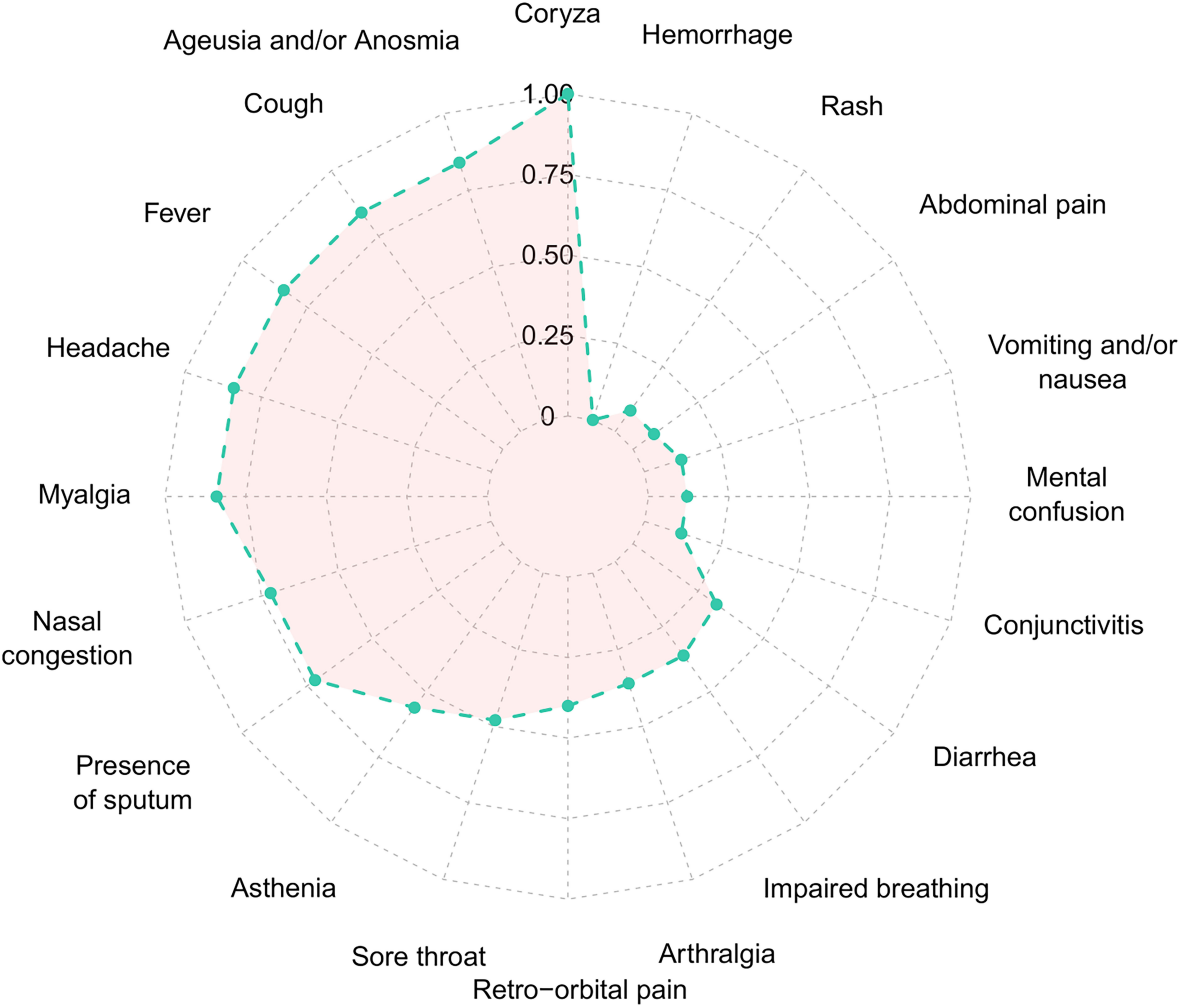
Frequency of each symptom of the Coronavirus Disease 2019 in the 38 SARS-CoV-2 positive patients. Normalized frequency considering the presence of the symptom with the lowest (*n* = 1) and highest (*n* = 26) value, to values between 0 and 1. SARS-CoV-2, severe acute respiratory syndrome coronavirus 2.

From February 2020 to the epidemiological week 45 (first week of November 2020), Brazil had intense SARS-CoV-2 dissemination characterized in the country as the first pandemic phase. In this period, we had monitored symptomatic individuals to characterize the SARS-CoV-2 infection status of the patients. Once positive on the molecular assay, we monitored the patients weekly by collecting NP-OP swabs and blood derivatives, such as whole blood, serum, and plasma. We found a positive correlation by the detection of Envelope and RdRp genes (*r* = 0.89) in the first sample collected in the molecular assay (Figure 1A). Thirty-eight mild symptomatic patients were followed weekly from the first episode of positivity until they completed at least two or three consecutive episodes of negativity by RT-qPCR in the NP-OP. The duration of the positivity after the onset of symptoms was not significantly different (*p*-value = 0.3531) between female (average 22.67 days ± SD 19.88) and male (average 33.34 ± SD 41.71) biological sexes (Figure 1B). The duration of symptoms in females (average 32 days ± SD 11.51) and males (average 34 days ± SD 18.14; Figure 1C) was similar (*p*-value = 0.8887). The length of time of the symptoms was weakly correlated with the length of the positivity (Figure 1D). Among the total number of surveyed patients, 7.9% (3/38) of them were RT-qPCR positive for an “atypical” period and were considered as “outliers” (Figure 1B), such as one female (71 days) and two male (81 and 232 days) individuals. The positive symptomatic patients presented a wide spectrum of mild-illness symptoms, the most frequent being coryza, ageusia and/or anosmia, cough, fever, headache, myalgia, nasal congestion, and presence of sputum (Figure 2).

**Figure 3:**
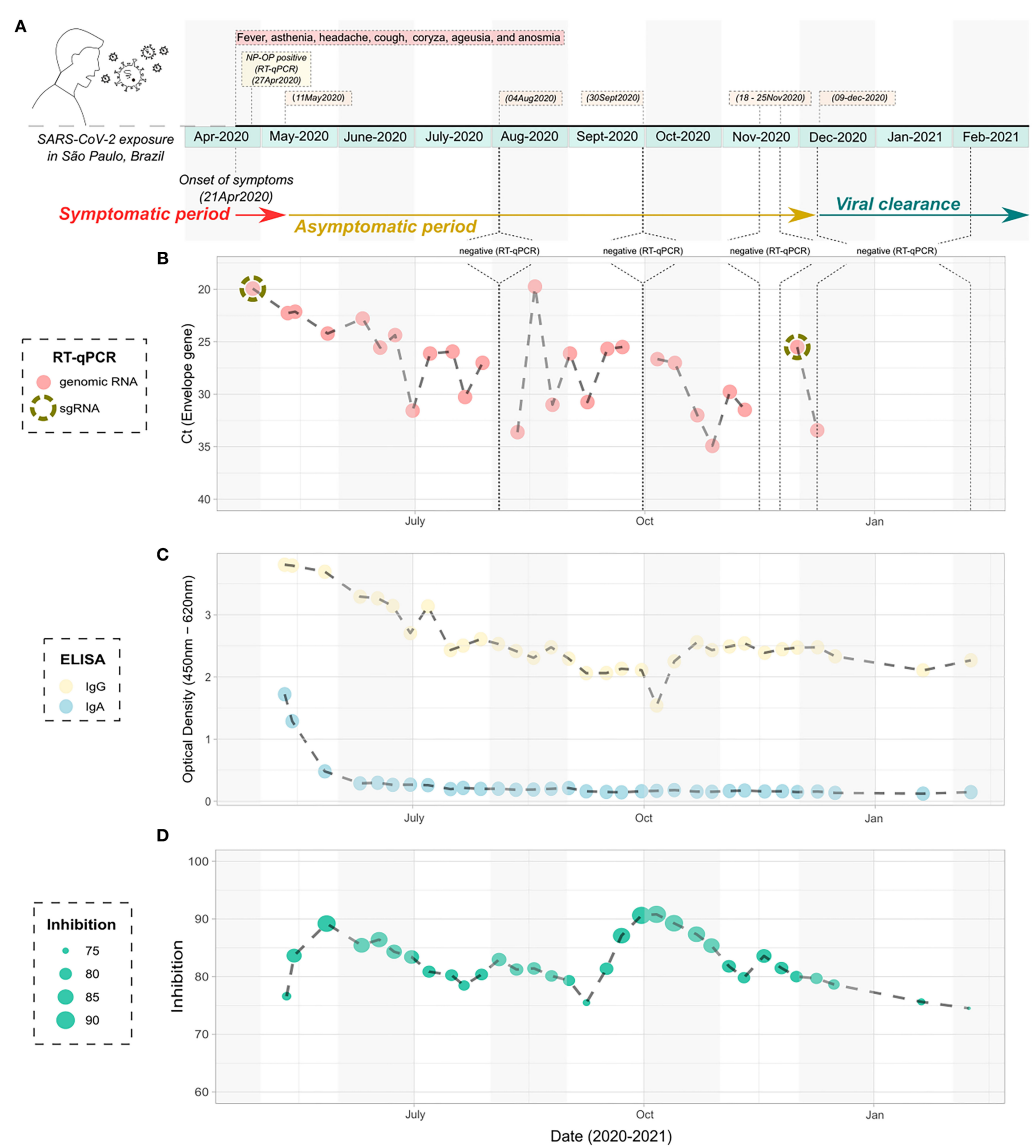
Timeline infection of the atypical studied patient with prolonged detection of viral RNA. **(A)** Schematic figure containing the diagnostic points, harvesting, and symptoms over time. **(B)** Cycle threshold to the Envelope gene of the SARS-CoV-2 according to the timeline of infection, as determined by RT-qPCR. The NP-OP swabs positive to the detection of subgenomic RNA are indicated by the dashed circle. **(C)** ELISA data showing the detection of IgA and IgG specific anti- SARS-CoV-2 nucleocapsid according to the timeline of infection. **(D)** Inhibition of the SARS-CoV-2/ACE2 ligation mediated by receptor-binding domain by neutralizing antibodies, according to the timeline of infection. SARS-CoV-2, severe acute respiratory syndrome coronavirus 2; NP-OP, nasopharyngeal-oropharyngeal; ELISA, enzyme-linked immunosorbent assay.

**Figure 4:**
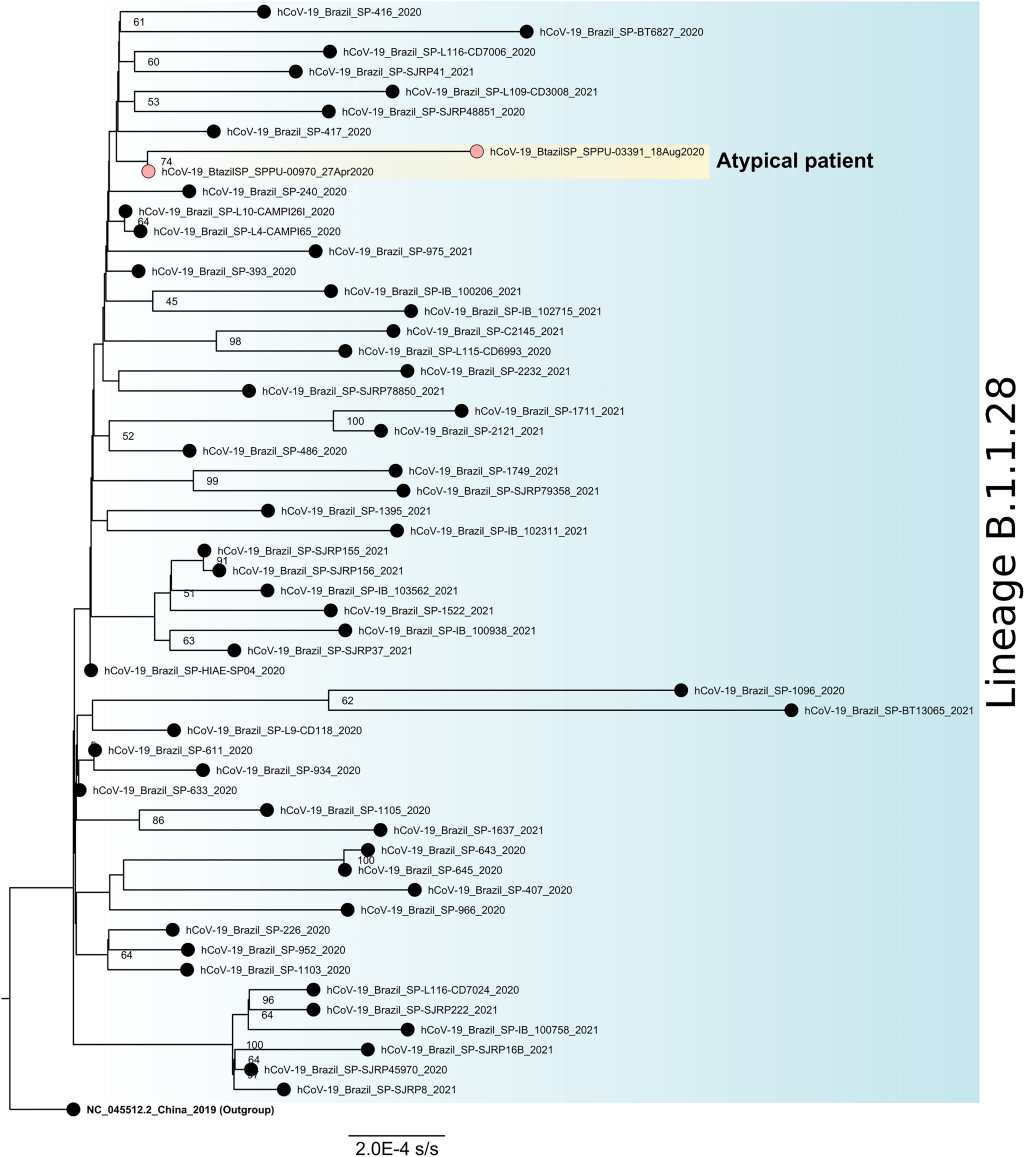
Maximum likelihood phylogenetic tree for SARS-CoV-2 lineage B.1.1.28 based on full-length genome sequences (*n* = 56). The sequence NC_045512.2 was used as an outgroup to reroot the tree and the nodes are labeled with bootstrap support values higher than 50. The two sequences obtained from the atypical patient are highlighted in yellow. SARS-CoV-2, severe acute respiratory syndrome coronavirus 2.

One of those outliers presented a very peculiar picture regarding positivity over time. The male patient, 38-year-old, was previously diagnosed with HIV without AIDS as confirmed by negative molecular diagnosis for HIV infection and normal levels of T CD4^+^ and CD8^+^ lymphocytes both before and after the infection with SARS-CoV-2 (Supplementary Table 2). The patient did not present any other comorbidity, started the clinical symptoms compatible with the classic condition caused by COVID-19 on April 21, 2020, notably fever, asthenia, headache, cough, coryza, ageusia, and anosmia until May 11, 2020 (Figure 3A). In total, the symptoms lasted for 20 days. The patient was diagnosed as positive to the SARS-CoV-2 by RT-qPCR on the seventh day after the onset of symptoms (Figure 3B), with the lineage B.1.1.28, as ascertained by sequencing (Figure 4). Up to 232 days after the onset of symptoms, the patient was still positive in the RT-qPCR assay for the presence of vRNA (Figure 3B). Furthermore, two of the collected samples were also positive for sgRNA, the first (April 27, 2020) and the one before the last sample (December 1, 2020) (Supplementary Figure 2), suggesting at least 224 days of viral replicative activity. Before the last positivity collection point, the patient had four negative harvestings in three moments of the infectious process, the last being for 2 consecutive weeks in November 2020 (Figure 3B). None of the samples was positive for viral isolation in cell culture.

Serological samples of this atypical patient were then tested in ELISA for the presence of anti-nucleocapsid (Nc) IgA and IgG antibody levels. We observed that specific anti-Nc IgA was detected from 3 weeks after the onset of symptoms (May 11, 2020) and persisted for the next two sera sample harvestings, (May 14, 2020 and May 27, 2020), remaining at basal levels until the last blood collection of the studied period (Figure 3C). On the other hand, anti-Nc IgG levels were detectable during the entire course of infection, with high OD values throughout the analyzed period, being the highest values observed in the first 2 months of the study, corresponding to the points with the greatest amount of vRNA (Figure 3B). Inhibition assays showed that total neutralizing antibody levels increased in the first 14–21 days of positivity for the molecular envelope gene assay, and around 85% of inhibition persisted during the sampling period (Figure 3D). In accordance, a perfect correlation was observed between the peaks of serum neutralizing antibodies and the decrease of the viral charge at the beginning of the asymptomatic period and the negative amplification of the envelope gene by RT-qPCR every since.

Among all NP-OP positive samples, the genomic monitoring was performed in three positive samples of this patient to perform genomic sequencing, one at the beginning (collection date = 27-Apr-2020), one at the middle (collection date = August 18, 2020), and one at the end (collection date = December 1, 2020) of the positivity period, all of them been chosen based on the combination of Ct values and period of infection (Figure 4). We obtained a complete genome for the first one, and for the other two samples only partial genomes. All sequences were characterized as lineage B.1.1.28. The last sequenced sample (collection date = December 1, 2020) had low coverage and was discarded from the phylogenetic analysis. The two sequences included in the phylogenetic studies were grouped in a monophyletic group when compared to other sequences isolated in São Paulo, Brazil for the same lineage and diversified between the first and the last atypical patient's harvestings, suggesting a within-host evolution of the SARS-CoV-2 (Figure 4).

## Discussion

The clinical manifestations induced by SARS-CoV-2 vary from a broad spectrum of symptoms, ranging from asymptomatic or mild-to-severe cases of COVID-19 (28–31). In autopsy investigations, the SARS-CoV-2 was identified in several organs, such as the lung, heart, and kidney (31). The viral infection has been associated with respiratory, gastrointestinal, hepatic, cardiac, renal, and neurological dysfunctions (30, 32), which leads to a multisystem inflammatory disease, acute respiratory distress syndrome, multiple organ failure, and death in severe cases (30, 32, 33). The dysfunctions induced by COVID-19 are manifested by signs and symptoms, which when well elucidated can be crucial to the clinical diagnosis and treatment (34). Initial descriptions of the average period of vRNA presence in samples of the patients who survived the infection are around 20 days, although other studies have identified atypical cases that can last up to 154 days (11, 12).

In the present study, the duration of positivity to SARS-CoV-2 by RT-qPCR was not statistically significantly different comparing biological sexes, with an average of 33.34 days for males and 22.67 days for females. However, one outlier was noted within the female group and two males. One male outlier patient remained detectable for SARS-CoV-2 for 232 days from the onset of symptoms. It is worth noting that a suggestive replicative viral activity observed in this particular patient was detectable at least until 224 days after the beginning of the infection, with lower Ct values even in advanced phases of the COVID-19 what may be of epidemiological significance in terms of transmission. This was clearly shown by the correlation between the detection of both sgRNA and the genomic envelope gene of SARS-CoV-2 (Supplementary Figure 2). Although the clinical and molecular conditions caused by viruses characterized as acute infections are generally quickly resolved, some of them can cause a long-lasting infection in mild (35) or severe conditions (36), and the impact in transmissibility and pathology still needs to be understood. Thus, it is very important to clarify whether a prolonged vRNA shedding is correlated to the clinical outcome of the infection, or yet to the condition of the patients, such as immunosuppressive states or occurrence of comorbidities.

The serological data of the atypical individual analyzed here above indicated that during the period of molecular vRNA detection, it was possible to detect specific IgA and IgG antibodies against SARS-CoV-2 Nc antigen. These data are compatible with the development of a humoral immune response raised against the virus very early after infection. Our results showed that both IgA and IgG anti-SARS-CoV-2 were already present in the first collected serum sample, 20 days after the onset of the symptoms. As with other viral infections (37, 38), the detection of serum IgA antibodies was limited in time, while the presence of IgG antibodies could be identified in the serum for long periods and present during the entire period of the study. Additionally, from the beginning of the sera collection, we identified the presence of total neutralizing antibodies (which can be of all immunoglobulin classes), with fluctuations during sampling. Despite an unrestrained innate immunity that can account for virus clearance at the beginning of acute infections (39), the specific responses observed here may reflect an early attempt of the immune system to control the viral infection by the induction of a robust neutralizing humoral immune response.

On top of the development of humoral and cellular immune responses, viruses escape from other immunological barriers imposed to clear the infection. Usually, to avoid an immune-mediated viral clearance, viruses frequently use a combination of several different strategies to subvert recognition by the immune system, such as (i) settling latent infections, (ii) replicating in immune-privileged sites, (iii) downregulating the expression of immune recognition signals on the surface of infected cells, or (iv) undergoing antigenic variation or else mechanisms for suppressing the immunological response (40). Possibly, a combination of one or more of these mechanisms could explain the prolonged viral shedding in the studied patient.

Nevertheless, the integration of the reverse-transcribed RNA into the genome of cultured human cells with the possibility of being expressed in tissue cells derived from humans seems to be an explanation, contradictorily proposed to clarify the persistent detection of SARS-CoV-2 RNA after COVID-19 recovery (41). This hypothesis does not seem to be the case of the outlier male patient studied here, since (i) we showed that sgRNA, a molecular marker for active SARS-CoV-2 replication (42, 43) was found during the long-term infection, (ii) there was a fluctuation in the levels of virus neutralization over time possibly characterizing the dynamics of the humoral immune response directed to the RBD antigen that binds to the human ACE2 receptor and yet, and (iii) the genetic divergence accumulated throughout the viral infection process (Figure 4) indicating an intra-host evolution over time. In addition, although the viral shedding was prolonged, the elimination of the viral infection was ultimately observed.

Conversely, since the outlier male patient studied here is seropositive for HIV since 2018, it could be hypothesized that this comorbidity would be impacting an appropriate immune response against SARS-CoV-2 through possible mechanisms of immunosuppression. It is worth noting that the differential lymphocytic markers to monitor HIV infection of this patient were consistently normal since the beginning of the antiretroviral therapy in 2019. Still, in-depth implications of HIV infection on the overall functioning of the immune system, even in treated individuals, are not fully comprehended (44), and data published elsewhere show that during the acute phase of HIV, there is a critical loss of memory CD4^+^ lymphocytes mainly in lymphoid tissues (45), which are critical to the maintenance of a fully competent immune system. The post-effects of this massive CD4^+^ loss may range to increased cell turnover and disrupted activation/differentiation and maturation of immune system cells, possibly due to indirect effects of the HIV replication (44, 46). Also, post-acute phase HIV causes a dramatic skewing of the lymphocyte population that is not fully recovered after effective antiretroviral treatment (47), possibly due to a decreased thymic functioning and HIV-induced lymph nodes architecture changes (48) that may impact innate and adaptive immune responses.

Thus, it remains to be concluded how the effects of HIV infection could be impacting positively or negatively the clearance of the SARS-CoV-2. In this sense, it is well recognized that an appropriate cell-mediated immune response mediated by the activation of CD4^+^ and CD8^+^ T cells producing class I interferon, i.e., interferon (IFN)-α and IFN-β, and the activation of B cells producing neutralizing antibodies are associated with a favorable clinical outcome of COVID-19 (49–52). However, other data have correlated HIV treatments to the increased incidence of diabetes mellitus, hyperglycemia, and drug-altered metabolism (53), known as rather enhancing the SARS-CoV-2 replication (54). Altogether, our data suggest that if the previously existing HIV-positive status is somehow related to a prolonged infection, it may instead be favoring the emergence of new variants.

Finally, since the severity of the clinical condition of patients with COVID-19 or the resistance status to the infection is previously shown to be associated with a genetic background of the host (55, 56), it could also be hypothesized that the prolonged viral shedding is related to the host genetic environment. Still, genetic factors associated with the SARS-CoV-2 viruses or specific for certain viral lineages could certainly influence viral shedding. Other linked factors to the host, such as age, other comorbidities association, nutritional state, and previous exposure to different pathogens, could interfere with such a differential behavior of the SARS-CoV-2 during the infectious process and affect healing and transmission.

## Data Availability Statement

The original contributions presented in the study are publicly available. This data can be found here: https://www.ncbi.nlm.nih.gov/genbank/, MW495017.

## Ethics Statement

The studies involving human participants were reviewed and approved by Human Research Ethics Committee of the Biomedical Sciences Institute—University of São Paulo (CEPSH-ICB-USP) (n°. 4.036.252). The patients/participants provided their written informed consent to participate in this study. Written informed consent was obtained from the individual(s) for the publication of any potentially identifiable images or data included in this article.

## Author Contributions

MC, AV, LG, AC, and PM: conceptualization. MC and SM: data curation. MC, AV, CM, SA, SM, VB, SB, LO, YA, and AC: formal analysis. PM: funding acquisition. MC and PM: project administration. MC, AV, CM, SA, SM, VB, SB, LO, YA, LG, AC, and PM: investigation, methodology, resources, and writing—review and editing. MC: software. JP and PM: supervision. MC, AV, SM, and PM: validation and writing—original draft. MC: visualization. All authors contributed to the article and approved the submitted version.

## Funding

This work was supported by the São Paulo Research Foundation (FAPESP—process #2017/27131-9), the Institut Pasteur (#DI2020-16/18), and the Cooperation and Cultural Action Services of the São Paulo French Consulate (#185BRA1184-−2020/SPPU). MC, AV, CM, SA, and VB received FAPESP Grants Nos. 2019/24518-5, 2019/20708-4, 2020/01487-4, 2017/23519-2, and 2018/11612-0, respectively. LO and YA received a Coordination for the Improvement of Higher Education Personnel (CAPES) Grant No. 88887.423542/2019-00 and 88887.474625/2020-00, respectively. JP is PI of a G4-grant from Institut Pasteur (#FUSP-3303-01). LG received a Brazilian National Council of Scientific and Technological Development (CNPq) Grant No. 152365/2019-2. AC was funded by Institut Pasteur/FUSP-Project #3303.

## Conflict of Interest

The authors declare that the research was conducted in the absence of any commercial or financial relationships that could be construed as a potential conflict of interest.

## Publisher's Note

All claims expressed in this article are solely those of the authors and do not necessarily represent those of their affiliated organizations, or those of the publisher, the editors and the reviewers. Any product that may be evaluated in this article, or claim that may be made by its manufacturer, is not guaranteed or endorsed by the publisher.
