# Supplementary material for: Atypical Prolonged Viral Shedding With Intra-Host SARS-CoV-2 Evolution in a Mildly Affected Symptomatic Patient
Source: Front Med (Lausanne). 2021 Nov 26;8:760170. doi: 10.3389/fmed.2021.760170 (PMC8661089; doi:10.3389/fmed.2021.760170)
Supplement: Supplementary file 1 [file Data_Sheet_1.docx]

**Supplementary information for:**

**Atypical prolonged viral shedding with intra-host SARS-CoV-2 evolution in a mildly affected symptomatic patient**

Marielton dos Passos Cunha^*^, Ana Paula Pessoa Vilela, Camila Vieira Molina, Stephanie Maia Acuña, Sandra Marcia Muxel, Vinícius de Morais Barroso, Sabrina Baroni, Lilian Gomes de Oliveira, Yan de Souza Angelo, Jean Pierre Schatzmann Peron, Luiz Gustavo Bentim Góes, Angélica Cristine de Almeida Campos, Paola Minóprio^*^

^*^ **Corresponding authors**:

marieltondospassos@gmail.com (MPC); paola.minoprio@pasteur.fr (PM)

**Contents**:

- Supplementary Figure 1
- Supplementary Figure 2
- Supplementary Table 1
- Supplementary Table 2


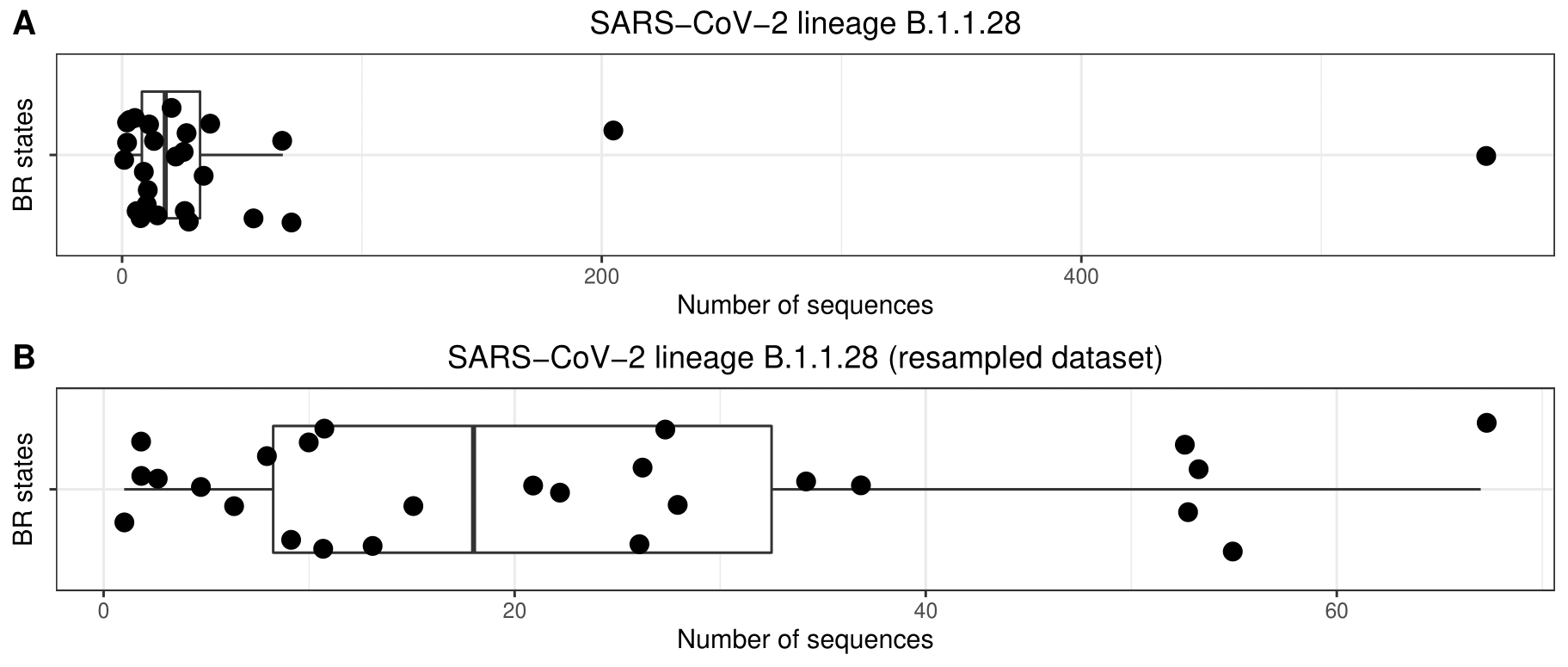


**Supplementary Figure 1**. The number of complete SARS-CoV-2 lineage B.1.1.28 genomes available in the GISAID platform until June/2021 isolated in Brazil. Boxplot represents the 75^th^ percentile, median, 25^th^ percentile, and the whiskers extend to the highest and lowest value in the 1.5 x interquartile range.


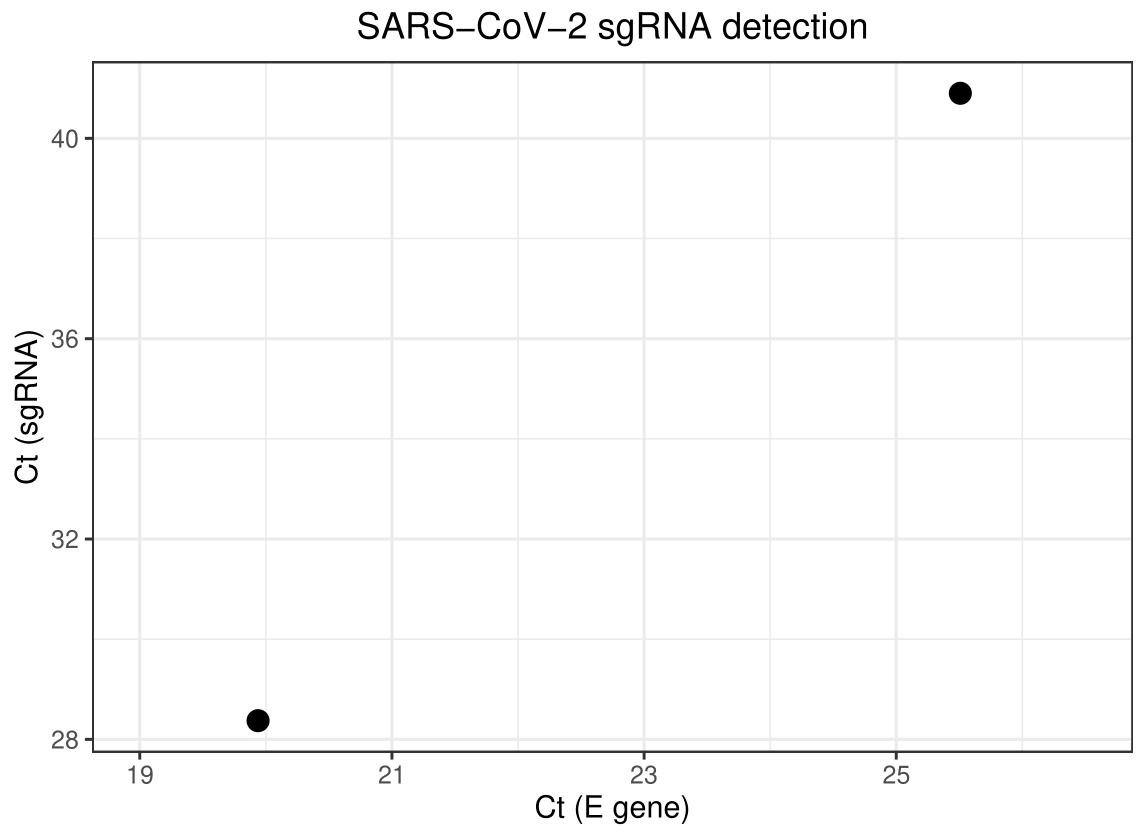


**Supplementary Figure 2**. SARS-CoV-2 subgenomic RNA detection.

**Supplementary Table 1**. Clinical and laboratory information from patients followed in the study (n = 38).

| **Sample ID** | **Biological sex** | **Age** | **Collection date** | **Envelope Ct** | **RdRp Ct** | **anti-RBD SARS-CoV-2 neutralizing antibodies** | **Onset of symptoms** | **End of symptoms** | **Last day of positivity** | **Days of symptoms** | **Days of positivity after symptom onset** |
| --- | --- | --- | --- | --- | --- | --- | --- | --- | --- | --- | --- |
| SPPU-00007 | F | 37 | 16-mar-20 | 19.22 | 26.338 | NA | 12-mar-20 | NA | 22-mai-20 | NA | 71 |
| SPPU-00021 | F | 27 | 18-mar-20 | 26.39 | 28.746 | NA | 5-mar-20 | NA | 7-abr-20 | NA | 33 |
| SPPU-00459 | M | 23 | 20-abr-20 | 23.345 | 26.52 | NA | 10-abr-20 | 14-jul-20 | 30-jun-20 | 95 | 81 |
| SPPU-00463 | M | 23 | 20-abr-20 | 23.733 | 26.653 | NA | 9-abr-20 | 3-jun-20 | 29-mai-20 | 55 | 50 |
| SPPU-00943 | M | 44 | 27-abr-20 | 28.800 | 31.464 | NA | 22-abr-20 | 16-mai-20 | 27-abr-20 | 24 | 5 |
| SPPU-00970 | M | 38 | 27-abr-20 | 19.938 | 23.564 | NA | 21-abr-20 | 11-mai-20 | 9-dez-20 | 20 | 232 |
| SPPU-01013 | M | 44 | 27-abr-20 | 22.761 | 26.309 | NA | 20-abr-20 | 11-mai-20 | 14-mai-20 | 21 | 24 |
| SPPU-01135 | M | 45 | 29-abr-20 | 22.483 | 27.426 | NA | 21-abr-20 | 16-mai-20 | 16-mai-20 | 25 | 25 |
| SPPU-01137 | M | 24 | 29-abr-20 | 23.923 | 28.294 | NA | 21-abr-20 | 29-mai-20 | 14-mai-20 | 38 | 23 |
| SPPU-01377 | F | 41 | 6-mai-20 | 26.905 | 30.633 | NA | 26-abr-20 | 22-mai-20 | 6-mai-20 | 26 | 10 |
| SPPU-01390 | F | 54 | 7-mai-20 | 34.248 | 35.658 | NA | 22-abr-20 | 7-mai-20 | 7-mai-20 | 15 | 15 |
| SPPU-01460 | M | 28 | 11-mai-20 | 28.190 | 29.358 | NA | 21-abr-20 | 27-mai-20 | 27-mai-20 | 36 | 36 |
| SPPU-01717 | M | 46 | 25-mai-20 | 14.058 | 17.152 | NA | 15-mai-20 | 7-jul-20 | 15-jun-20 | 53 | 31 |
| SPPU-01718 | F | 48 | 25-mai-20 | 16.484 | 19.534 | NA | 21-mai-20 | 7-jul-20 | 3-jun-20 | 47 | 13 |
| SPPU-02019 | M | 45 | 3-jun-20 | 16.627 | 19.772 | NA | 30-abr-20 | 10-jun-20 | 17-jun-20 | 41 | 48 |
| SPPU-02023 | F | 31 | 3-jun-20 | 27.085 | 28.165 | NA | 19-mai-20 | 17-jun-20 | 3-jun-20 | 29 | 15 |
| SPPU-02025 | M | 37 | 3-jun-20 | 34.920 | - | Positive | 24-mai-20 | 10-jun-20 | 3-jun-20 | 17 | 10 |
| SPPU-02026 | F | 43 | 3-jun-20 | 35.900 | - | Positive | 8-mai-20 | 23-jun-20 | 3-jun-20 | 46 | 26 |
| SPPU-02363 | F | 43 | 23-jun-20 | 24.255 | 25.171 | NA | 8-jun-20 | 14-jul-20 | 23-jun-20 | 36 | 15 |
| SPPU-02375 | F | 49 | 23-jun-20 | 21.253 | 23.581 | NA | 17-jun-20 | 14-jul-20 | 23-jun-20 | 27 | 6 |
| SPPU-02644 | M | 54 | 7-jul-20 | 34.933 | - | * | 23-jun-20 | 21-jul-20 | 4-ago-20 | 28 | 42 |
| SPPU-02676 | M | 59 | 14-jul-20 | 35.996 | - | ** | 6-jun-20 | 21-jul-20 | 14-jul-20 | 45 | 38 |
| SPPU-02696 | M | 31 | 14-jul-20 | 35.444 | - | Positive | 5-jul-20 | 28-jul-20 | 14-jul-20 | 23 | 9 |
| SPPU-02707 | M | 36 | 14-jul-20 | 37.044 | - | *** | 7-jul-20 | 11-ago-20 | 28-jul-20 | 35 | 21 |
| SPPU-02777 | M | 29 | 21-jul-20 | 33.750 | - | * | 13-jul-20 | 15-set-20 | 25-ago-20 | 64 | 43 |
| SPPU-02787 | M | 27 | 21-jul-20 | 32.206 | - | Positive | 16-jul-20 | 28-jul-20 | 21-jul-20 | 12 | 5 |
| SPPU-02873 | M | 25 | 28-jul-20 | 33.036 | - | Negative | 28-jun-20 | 28-jul-20 | 28-jul-20 | 30 | 30 |
| SPPU-02875 | M | 22 | 28-jul-20 | 32.707 | 39.301 | NA | 24-jul-20 | 11-ago-20 | 28-jul-20 | 18 | 4 |
| SPPU-02978 | M | 70 | 4-ago-20 | 12.410 | 18.301 | NA | 1-ago-20 | 15-set-20 | 1-set-20 | 45 | 31 |
| SPPU-03061 | M | 25 | 4-ago-20 | 16.541 | 19.517 | NA | 16-jul-20 | 11-ago-20 | 18-ago-20 | 26 | 33 |
| SPPU-03065 | M | 31 | 4-ago-20 | 34.333 | 31.465 | NA | 20-jul-20 | 18-ago-20 | 4-ago-20 | 29 | 15 |
| SPPU-03100 | M | 51 | 6-ago-20 | 26.077 | 25.523 | NA | 29-jul-20 | 15-set-20 | 18-ago-20 | 48 | 20 |
| SPPU-03101 | M | 47 | 6-ago-20 | 17.160 | 17.732 | NA | 30-jul-20 | 8-set-20 | 25-ago-20 | 40 | 26 |
| SPPU-03112 | M | 26 | 6-ago-20 | 24.875 | 27.665 | NA | 1-ago-20 | 15-set-20 | 1-set-20 | 45 | 31 |
| SPPU-03114 | M | 28 | 6-ago-20 | 33.080 | 25.15 | NA | 3-ago-20 | 25-ago-20 | 18-ago-20 | 22 | 15 |
| SPPU-03559 | M | 42 | 25-ago-20 | 32.503 | 33.288 | NA | 22-ago-20 | 25-ago-20 | 1-set-20 | 3 | 10 |
| SPPU-03568 | M | 20 | 25-ago-20 | 23.603 | 25.867 | NA | 18-ago-20 | 15-set-20 | 8-set-20 | 28 | 21 |
| SPPU-03574 | M | 52 | 25-ago-20 | 16.325 | 19.484 | NA | 17-ago-20 | 8-set-20 | 25-ago-20 | 22 | 8 |

* RT-qPCR positive for envelope gene in 5 consecutive weeks and negative for RdRp gene. The patient was considered as bearing an inconclusive diagnosis due to the detection of the Envelope gene alone, with high Ct data.

** Previously diagnosed positive for COVID-19 by an external laboratory.

*** RT-qPCR positive for envelope in 2 consecutive weeks, but only positive to RdRp in the second week, confirming the diagnosis.

**Supplementary Table 2**. Monitoring data of the atypical SARS-CoV-2 patient seropositive for the Human Immunodeficiency virus infection. T CD4^+^ reference range (RR) = ≥ 300 cells/mm^3^; T CD8^+^ RR = ≥ 300 cells/mm^3^; CD4^+^/CD8^+^ ratio = 1; CD45^+^ (monocytes + lymphocytes) RR = ≥ 500 cells/mm^3^ (1–3).

| **Collection date** | **Number of viral copies/ml** | **Detection method** | **T CD4^+^ cells** | **T CD8^+^ cells** | **CD4^+^/CD8^+^ ratio** | **CD45^+^** |
| --- | --- | --- | --- | --- | --- | --- |
| 17-dec-2018 | 142867 | RT-qPCR | 826 | 1588 | 0.52 | 3098 |
| 22-apr-2019 | not detected | RT-qPCR | 1034 | 1382 | 0.74 | 3209 |
| 20-aug-2019 | not detected | RT-qPCR | 1016 | 1206 | 0.84 | 2995 |
| 14-jan-2020 | not detected | RT-qPCR | 663 | 748 | 0.88 | 1940 |
| 09-jun-2020 | not detected | RT-qPCR | NA | NA | NA | NA |
| 13-oct-2020 | not detected | RT-qPCR | 967 | 976 | 0.99 | 2754 |
| 05-apr-2021 | not detected | RT-qPCR | 1060 | 1168 | 0.90 | 3049 |

**References**

1. Organization WH. HIV/AIDS. Available at: https://www.who.int/health-topics/hiv-aids#tab=tab_1

2. Mussini C, Lorenzini P, Cozzi-Lepri A, Lapadula G, Marchetti G, Nicastri E, Cingolani A, Lichtner M, Antinori A, Gori A, et al. CD4/CD8 ratio normalisation and non-AIDS-related events in individuals with HIV who achieve viral load suppression with antiretroviral therapy: an observational cohort study. *Lancet HIV* (2015) **2**:e98–e106. doi:10.1016/S2352-3018(15)00006-5

3. Koyalta D, Jenabian M-A, Nadjiouroum N, Djouater B, Djemadji-Oudjeil N, Ndjoyi-Mbiguino A, Bélec L. Single-platform, volumetric, CD45-assisted pan-leucogating flow cytometry for CD4 T lymphocytes monitoring of HIV infection according to the WHO recommendations for resource-constrained settings. *BMC Res Notes* (2013) **6**:1–10. doi:10.1186/1756-0500-6-169
